# Cases from the Practice of Dr. Hansen

**Published:** 1890-09

**Authors:** 


### CASES FROM THE PRACTICE OF DR. OSCAR HANSEN, COPENHAGEN.

ALLG. HOM. ZEITUNG 23, '90.

1. E. L., thirty-four years old, single, suffered formerly from ulcus ventriculi and was always anæmic. Ailing for four weeks, she complains of heaviness in head, vertigo, buzzing in ears, hebetude, and sleepiness. Pressure and burning in pit of stomach, with vomiting of flat water, always between meals. Thirst, drinks little and often. Stool soft, the discharge is yellowish-white mixed with mucus, three and four times in twenty-four hours. Copious menses every two weeks. Frequent nocturnal urination, but only a small quantity passed. Urine light-yellow, foaming, clear, and acid; specific gravity 1014, containing five per cent. albumen and cylinders; passes only half a litre in twenty-four hours; œdema of eyelids and around malleoli, mucous membranes pale. Sounds of heart somewhat blowing. Phosphor.$^{3c}$, three drops, three times daily. September

10th (three weeks later).—Bowels normal, œdema gone, no other change. Arsen.$^{3c}$. September 30th.—Headache, vertigo, the pains in the pit of the stomach, the vomiting, and the lassitude all gone. Passes one litre urine in twenty-four hours, and does not get up at night to urinate. Menses after twenty-four days and less copious. Two per cent. albumen in the urine, no cylinders. November 15th.—Urine normal and would be discharged cured.

(The differentiation between Phosphorus and Arsenicum in Bright's disease Buchner has well given in his little work on *Morbus Brightii*, where he clearly shows that Phosphorus is for the venous circulation (right heart) what Arsenicum is for the arterial circulation (left heart). The patient had suffered from ulcus pepticum, an affection not rare in chlorotic women, and both remedies may be indicated for that ulcer, but while we find in Phosphorus pressure in stomach after eating, with *immediate* vomiting of food (she vomited between meals) or even after a swallow of water, with frequent fainting, cold extremities, etc., we read of Arsenicum: Gagging, nausea, and vomiting, mostly after two hours, even from the lightest kind of food; stomach tender to the slightest touch; frequent vomiting, with fear of death. In morbus Brightii Phosphorus is more suitable to chronic cases with their degenerative character, while Arsenic suits more primary cases with puffiness of eyes and swelling of feet with exhausting diarrhœa and cardiac dyspnœa. Arsenic is also the remedy for menorrhagic chlorosis, while the tubercular (psoric) diathesis prevails in Phosphorus, which was here not indicated, while Arsenicum covered every indication.)

2. A Case of Chorea. L. M., twelve years old, the son of a laborer, enjoyed good health, when two months ago he was attacked with chorea. Involuntary twitchings and motions over the whole body, restless in daytime and easily frightened at night, when he twitches in his face. His motions are awkward, especially when dressing or undressing; speech unintelligible. Looks pale, though his appetite is good. February 10th.—Cuprum-met.$^{6c}$ five drops, three times daily. February 21st.—No change. March 2d.—Worse all over; twitchings, followed by great lassitude: loss of memory, idiotic features, must be fed. Stramonium$^{3c}$ three times daily. March 21st.—Great improvement, is able to feed himself, speaks distinctly, and is more lively. April 25th.—All choreic symptoms gone, and as a constitutional improver he took now Calcarea-phos.$^{30c}$ three drops, morning and evening, and about the middle of May he was discharged.

Krewssler, forty years ago, in a condensed little book on homœopathic treatment, considers Stramonium nearly the chief remedy for chorea, as it is apt to cause extreme mobility of the limbs; it has been known for years past that the vapors of Stramonium cause chorea. Of nearly equal importance he considers Hyoscyamus and Veratrum-album. We feel sorry that Hansen fails to record where the chorea began and what was the emotional or somatic (helminthiasis) cause of it. It is well known of Cuprum that its spasms are apt to begin in the fingers and toes and then spread all over the body, with excessive dyspnœa and threatening suffocatory attacks, hence the livor, followed by collapse. Under Stramonium, the fool's remedy, all movements are characterized by great violence, and finally may lead to idiocy; the harder the case from the start the more Stramonium is indicated, though the late Hempel, as usual recommends for such severe cases his beloved Aconitum napellus, while we rather think of Veratrum-viride in chorea magna, where the spasms keep up all night with nearly the same intensity. We have done well in such severe cases with Laurocerasus or Tarentula-hispanica, but where the duration of the disease tends to mental degradation, we would certainly have more confidence in Stramonium than in any other remedy. The cause of chorea is often hard to detect, we know only its effects, and the peculiar symptoms are often hard to find out. It racks terribly the whole nervous system, and constitutional antipsoric treatment may be necessary from the start, and is certainly indicated, to restore the equilibrium when the storm has passed away.

3. D. B., twenty-three years old, single, passed through scarlatina when a child and is now complaining for the last three months. Before her menses and on the first day of the flow severe pressing and lancinating pains in the left groin. Her diarrhœa is worst in the morning, frequently with œdema of the upper eyelids. Urine normal, appetite and sleep good. Apis-mellifica$^{3d}$, five drops four times daily, removed the diarrhœa and the dysmenorrhœa.

Though Apis acts more on the right ovary, we see it here acting equally well in left ovarian neuralgia; in fact, we meet among its symptoms cutting in left then in right ovary, paroxysmal, extending into thigh, worse while stretching. Hansen fails to tell us the character of the menstrual discharge, nor does he say anything about the character of the stools. It is a pity that reporters of cases fail so often to give us the peculiarities of a case, in that it might be used as a verification. At any rate, it verifies the morning diarrhœa, and as there is nothing said about pain, we may consider it a painless one, probably the intestinal muscles felt weakened by the ovarian pains preceding the flow for a week. Even that adjective "mornings" ought to be more specified, whether in bed or after rising, profuse or scanty.

4. A child of thirteen months had whooping-cough seven months ago, during which the present state developed, for which she was treated at the hospital without relief. March 2d, Hansen found: After a strong piping scream during the inspiration, breathing stops suddenly. The child turns pale in the face, cyanosis of the upper lips and nose, twitching of the extremities alternating with stiffness; cold sweat on the scalp, vomiting of mucus after the attack, which lasts three or four minutes and happens five or six times during the day, never at night. No other abnormality could be detected. Cuprum-met.$^{6c}$, three times daily, for the last two weeks, diminished the number of the attacks, but that was all. On account of the strong piping or whistling inspiration Iodium$^{2c}$, three drops three times daily, was prescribed, with partial benefit. April 10th.—Bronchitis set in, relieved by Aconite, Bryonia, and Phosphorus, and then on May 3d Iodium was again given and the attack gradually disappeared. Calcarea-phosphorica was then given for the troubles of dentition with copious perspiration of the head. It passed well through the summer and now leaves nothing to be desired.

The Halogens always will remain our stand-by in the treatment of laryngismus stridulus, but they have inspiration unimpeded and natural, expiration nearly impossible; on account of the whooping-cough my choice would have been Mephites, which has inspiration difficult and expiration also with cyanosis. Was the child psoric, scrofulous, and rachitic? for only thus can we explain the favorable action of Iodium.

